# Group VIB Calcium-Independent Phospholipase A_2_ (iPLA_2_γ) Regulates Platelet Activation, Hemostasis and Thrombosis in Mice

**DOI:** 10.1371/journal.pone.0109409

**Published:** 2014-10-14

**Authors:** Emiko Yoda, Kohmi Rai, Mai Ogawa, Yuki Takakura, Hiroshi Kuwata, Hidenori Suzuki, Yoshihito Nakatani, Makoto Murakami, Shuntaro Hara

**Affiliations:** 1 Division of Health Chemistry, Department of Healthcare and Regulatory Sciences, School of Pharmacy, Showa University, Shinagawa-ku, Tokyo, Japan; 2 Division of Morphological and Biomolecular Research, Graduate School of Medicine, Nippon Medical School, Bunkyo-ku, Tokyo, Japan; 3 Lipid Metabolism Project, Tokyo Metropolitan Institute of Medical Science, Setagaya-ku, Tokyo, Japan; National Cerebral and Cardiovascular Center, Japan

## Abstract

In platelets, group IVA cytosolic phospholipase A_2_ (cPLA_2_α) has been implicated as a key regulator in the hydrolysis of platelet membrane phospholipids, leading to pro-thrombotic thromboxane A_2_ and anti-thrombotic 12-(*S*)-hydroxyeicosatetranoic acid production. However, studies using cPLA_2_α-deficient mice have indicated that other PLA_2_(s) may also be involved in the hydrolysis of platelet glycerophospholipids. In this study, we found that group VIB Ca^2+^-independent PLA_2_ (iPLA_2_γ)-deficient platelets showed decreases in adenosine diphosphate (ADP)-dependent aggregation and ADP- or collagen-dependent thromboxane A_2_ production. Electrospray ionization mass spectrometry analysis of platelet phospholipids revealed that fatty acyl compositions of ethanolamine plasmalogen and phosphatidylglycerol were altered in platelets from iPLA_2_γ-null mice. Furthermore, mice lacking iPLA_2_γ displayed prolonged bleeding times and were protected against pulmonary thromboembolism. These results suggest that iPLA_2_γ is an additional, long-sought-after PLA_2_ that hydrolyzes platelet membranes and facilitates platelet aggregation in response to ADP.

## Introduction

Platelets play a key role in hemostasis through their ability to respond to vascular injury. When circulating platelets are exposed to collagen-rich subendothelium at the site of a vascular injury, platelets become activated, release granule contents, and generate thrombin and the lipid mediator thromboxane A_2_ (TXA_2_) [1], [2]. Secreted adenosine diphosphate (ADP), serotonin, and TXA_2_ amplify the initial stimulus in a positive feedback activation of platelets. In addition, α-granule proteins, such as P-selectin, which mediate adhesive interactions between platelets, leukocytes, and endothelial cells, play a pivotal role in the pathogenesis of thrombosis and inflammation [2], [3]. TXA_2_ is a potent platelet agonist and an arachidonic acid (AA) metabolite, produced via the cyclooxygenase (COX) pathway [4], [5]. Another platelet-derived lipid mediator, 12(*S*)-hydroxyeicosatetraenoic acid (12*(S)*-HETE), is also an AA metabolite produced via platelet-type 12-lipoxygenase and acts as a platelet antagonist [6], [7]. TXA_2_ formation is rapid and quickly reaches a plateau in activated platelets, whereas 12*(S)*-HETE formation is slower and continues to increase over a longer period of time.

AA is released from the *sn*-2 position of glycerophospholipids by the action of phospholipase A_2_ (PLA_2_). PLA_2_ enzymes have been classified into six major families: secretory PLA_2_ (sPLA_2_), cytosolic PLA_2_ (cPLA_2_), Ca^2+^-independent PLA_2_ (iPLA_2_), platelet-activating factor acetylhydrolases, lysosomal PLA_2_s and adipose-specific PLA_2_; each family occurs as multiple isoforms [8]. Platelets are known to contain both cPLA_2_α (also known as group IVA PLA_2_), a cPLA_2_ enzyme that requires micromolar concentrations of intracellular Ca^2+^ for translocation to membrane phospholipids, and group IIA sPLA_2_ (sPLA_2_-IIA), an sPLA_2_ enzyme that requires millimolar Ca^2+^ concentrations for its enzymatic activity [8]. AA production in platelets is dependent on cPLA_2_α but not on sPLA_2_-IIA [9]. A functional deficiency of cPLA_2_α diminished platelet aggregatory and secretory responses to collagen [10]. cPLA_2_α-deficient mice have prolonged bleeding times and are resistant to thromboembolism induced by injection of a mixture of ADP and collagen, indicating a role of this enzyme in platelet adhesive and hemostatic functions. However, residual AA release and TXA_2_ production were still observed in collagen- or ADP-stimulated platelets isolated from cPLA_2_α/sPLA_2_-IIA double-deficient mice. Furthermore, bromoenol lactone (BEL), an iPLA_2_ inhibitor, inhibits AA production in 12-*O*-tetradecanoylphorbol-13-acetate (PMA)- or thrombin-stimulated platelets [11], [12]. These reports have suggested that another PLA_2_ enzyme, possibly BEL-sensitive iPLA_2_ enzyme(s), may compensate for platelet activation.

To date, nine members of the iPLA_2_ family, also referred to as the patatin-like phospholipase-domain containing (PNPLA) family, have been identified. These iPLA_2_ isoforms have one or more nucleotide-binding motif (GXGXXG) and a lipase consensus site (GXSXG) separated by a 10–40-amino acid residue spacer linkage [13], [14]. Unlike cPLA_2_s and sPLA_2_s, iPLA_2_s do not require intracellular Ca^2+^ for enzymatic activity or membrane binding, and they are sensitive to BEL [15]–[17]. Among iPLA_2_s, it is assumed that two abundant isoforms –iPLA_2_γ/PNPLA8 (group VIB) and iPLA_2_β/PNPLA9 (group VIA)– serve as housekeeping enzymes responsible for phospholipid acyl group turnover and generation of the lysophospholipids necessary for AA incorporation [14], [18], [19].

Recently, several studies have revealed the role of iPLA_2_γ in lipid mediator production. For example, overexpression of iPLA_2_γ has been shown to promote spontaneous and agonist-stimulated release of AA, which is converted to prostaglandin E_2_ (PGE_2_) with preferred COX-1 coupling in HEK293 cells [20]. The induction of group IIA sPLA_2_ by pro-inflammatory stimuli has been shown to require iPLA_2_γ through production of certain lipid metabolite(s) in rat fibroblastic 3Y1 cells [21]. iPLA_2_γ could produce 2-arachidonoyl-lysophosphatidylcholine, a presumptive lipid mediator, through its PLA_1_ action [22]. In addition, disruption of the iPLA_2_γ gene in mice reduced the levels of prostaglandin F_2α_ (PGF_2α_) and D_2_ (PGD_2_) in skeletal and heart muscle and those of TXA_2_ in heart muscle [23]. Moreover, Ca^2+^-induced myocardial activation of iPLA_2_γ and the attendant release of AA and its metabolites, were attenuated by genetic ablation of iPLA_2_γ [24]. These results raise the possibility that iPLA_2_γ may be involved in AA release from glycerophospholipids in activated platelets.

In the current study, we investigated the role of iPLA_2_γ in platelets using iPLA_2_γ knockout (iPLA_2_γ-KO) mice. Our findings demonstrate that lack of iPLA_2_γ expression *in vivo* increased bleeding time and protected mice from thromboembolism. In studies using isolated platelets, iPLA_2_γ-KO mouse platelets were aggregated only poorly, and produced a reduced level of TXA_2_ in response to ADP. Furthermore, electrospray ionization mass spectrometry (ESI-MS) analysis of platelet phospholipids suggested that iPLA_2_γ mainly catalyzed the hydrolysis of AA-containing plasmalogen-type phosphatidylethanolamine (PE) and phosphatidylglycerol (PG) and subclasses in activated platelets. These results indicate that, together with cPLA_2_α, iPLA_2_γ plays a role in AA mobilization from specific AA-containing phosholipid pools in activated platelets.

## Materials and Methods

### Antibodies and Reagents

The study used iPLA_2_γ-KO mice on a C57BL/6j background, as described in a previous study [23]. All procedures involving animals were approved by the Institutional Animal Care and Use Committees of Showa University. ADP, prostaglandin E_1_ (PGE_1_), thrombin and anti-β-actin monoclonal antibody were purchased from Sigma (St Louis, MO). U46619 and AA were from Cayman Chemical (Ann Arbor, Michigan). Collagen reagent Horm (native collagen fibrils from equine tendons) was purchased from Nycomed Arzneimittel (Munchen, Germany). MRS2365 and MRS2279 were purchased from TOCRIS bioscience (Bristol, UK). Phosphatidylcholine (PC) with C_28∶0_, PE with C_28∶0_ and PG with C_28∶0_ were from Avanti Polar Lipids, Inc. (Alabaster, AL). Paraformaldehyde, glutaraldehyde, EPON, and uranyl acetate were obtained from TAAB Laboratories (Aldermaston, West Berkshire, UK). Rabbit anti-adenylyl cyclase (AC), phosphodiesterase (PDE) 3A and PDE5 polyclonal antibody, anti-cPLA_2_α monoclonal antibody and goat anti-COX-1 and G_αi_ antibody were purchased from Zymed Laboratories (South San Francisco, CA). Rabbit anti-iPLA_2_γ polyclonal antibody was prepared as described in previous studies [21], [23].

### Isolation of Platelets

Mice anesthetized with diethyl ether were used for cardiac puncture. The heart was exposed and a 1-ml syringe with a 25-gauge needle containing 100 µl of 3.8% (w/v) trisodium citrate was used to obtain about 1 ml of blood. The platelet-rich plasma (PRP) was obtained by centrifugation of whole blood at 250×*g* for 10 min at room temperature, platelet-poor plasma (PPP) was obtained by centrifugation of lower-phase blood at 800×*g* for 15 min at room temperature, and PRP were diluted by PPP at a concentration of 200×10^3^/µl for ADP, MRS2365 or MRS2279 stimulation. For ADP, collagen, thrombin, PMA, AA or A23187 stimulation, platelets were isolated by differential centrifugation from PRP, then were suspended in HEPES/tyrode’s (H/T) buffer (pH 7.35) [138 mM NaCl, 2.8 mM KCl, 3.75 mM NaH_2_PO_4_·12H_2_O, 0.8 mM MgCl_2_, 10 mM HEPES, 5.6 mM dextrose, 0.35% (w/v) bovine serum albumin], supplemented with 1 µM PGE_1_. Platelet suspension was incubated for 15 min at 37°C and centrifuged at 800×*g* for 15 min at room temperature. Final platelet suspension was adjusted to 200×10^3^/µl with H/T buffer without PGE_1_.

### Platelet Aggregation

Platelet aggregation (180 µl samples) was assessed in an aggregometer (HEMA tracer, LMS Co., Ltd., Tokyo, Japan) with constant stirring (100 rpm) at 37°C. The platelets were then incubated with various inhibitors, and without stirring, at 37°C, for various periods of time before agonists were added: collagen (1 µg/ml), ADP (10 µM), U46619 (5 µM), thrombin (0.1 U/ml), A23187 (5 µM), AA (100 µM), PMA (10 nM), MRS2365 (10 µM) and MRS2279 (10 µM). Aggregation was measured and expressed as a percent change in light transmission, with the value for blank sample (PPP or H/T buffer without platelets) set at 100%.

### SDS-PAGE and Immunoblotting

Ten-µg protein was subjected to SDS-PAGE using 7.5% or 12% gels under reducing conditions. The separated proteins were electroblotted onto nitrocellulose membranes (Schleicher & Schuell, Dassel, Germany) with a semidry blotter (Bio-Rad Laboratories, Hercules, USA) according to the manufacturer’s instructions. After blocking with 5% (w/v) skim milk in 10 mM Tris-HCl, pH 7.4, containing 150 mM NaCl and 0.05% Tween 20, membranes were probed with the respective antibodies (1∶5,000 dilution for iPLA_2_γ COX-1, P2Y_1_, P2Y_12_, AC, PDE3A, PDE5 and G_αi_; 1∶10,000 dilution for cPLA_2_α and β-actin) for 1 h, then incubated with horseradish peroxidase-conjugated anti-rabbit (1∶5,000 for iPLA_2_γ P2Y_1_, P2Y_12_, AC, PDE3A and PDE5) IgG, peroxidase-conjugated anti-goat (1∶5,000 for COX-1 and G_αi_) IgG and peroxidase-conjugated anti-mouse (1∶10,000 for cPLA_2_α and β-actin) IgG. After washing, the membranes were visualized with Western Lightning Chemiluminescence Reagent Plus (Perkin Elmer Life Sciences, Boston, MA, USA).

### Reverse Transcription PCR

Total RNA was extracted from mouse platelets with TRIzol reagent (Invitrogen Life Technologies, Carlsbad, CA, USA). First-strand cDNA synthesis was conducted using the SuperScript III reverse transcriptase kit (Invitrogen Life Technologies) according to the manufacture’s instructions. Five µg of total RNA in reaction mixture was primed with oligo (dT) (12–18 mer) primer (Invitrogen Life Technologies) to obtain cDNA. Then, 1 ml of the synthesized cDNA was used as the template for the mRNA amplification reactions. The PCR conditions were 96°C for 5 min, then 35 cycles of 96°C and 63°C for 30 s, and finally 68°C for 2 min on a thermal cycler (Applied Biosystetms). The reverse transcription PCR products were analyzed by 1% agarose gel electrophoresis with ethidium bromide. The primer pairs were *Pla2g4a* (forward: 5′- gcatggcactgtgtgatcag-3′, reverse: 5′-cgtgaagagaggcaaaggaca-3′); *Pla2g6* (forward: 5′-gcaaacactggcactctccaag-3′, reverse: 5′-cggagaatgactccaaatctgg-3′); *Pnpla8* (forward: 5′-gagactgccttccattacgct-3′, reverse: 5′-tcgtttggggtgtccacttc-3′).

### Electron Microscopy

The platelets suspended in H/T buffer were fixed by mixing with an equal volume of 2% glutaraldehyde in 0.1 M phosphate buffer (PB, pH 7.4) for 30 min at room temperture. The fixed cells were transferred to eppendorf tubes, then centrifuged at 2,000 rpm for 5 min at 4°C. The platelet pellets were dissected into blocks of 1-mm cubes, washed 5 times in 0.1 M PB, post-fixed with 1% osmium tetroxide in the same buffer for 1 h at 4°C, dehydrated with a graded ethanol series, and then embeded in Epon 812, according to the conventional method. Ultra-thin sections were cut with a diamond knife and stained with uranyl acetate and lead citrate, then examined with a JEM-1200EX electron microscope (JEOL, Tokyo, Japan) at an accelerating voltage of 80 kV.

### Measurement of ATP and Serotonin Secretion

After the reaction, platelets were removed by centrifugation in the presence of 5 mM of ice cold EDTA and 10 µg/ml indomethacin. The amounts of adenosine triphosphate (ATP) and serotonin in platelet-free supernatant fraction was measured using an ATP bioluminescence assay kit CLS II (Roche Applied Science, Mannheim, Germany) and EIA Serotonin kit (IMMUNOTECH SAS, Marseille, France), respectively, according to the manufacturer’s protocol.

### Ca^2+^ Influx

Washed platelets were loaded with fura-2 by incubation in RPMI1640 medium containing 5 µM fura-2/AM (Dojindo Laboratories, Kumamoto, Japan), PGE_1_ and 10% fetal bovine serum at 37°C for 40 min. The fura-2-loaded platelets were washed with H/T buffer (pH 7.35) containing PGE_1_ twice and resuspended in loading solution (145 mM NaCl, 10 mM HEPES, 10 mM MgCl_2_, 6 mM glucose, 5 mM KCl (pH 7.35)) at a concentration of 200×10^3^/µl, then activated with 10 µM ADP. Fluorescence was continuously recorded using CAF-110 (JASCO Co., Ltd., Tokyo, Japan) at 37°C by alternating the excitation wavelength between 340 and 380 nm, and detecting the fluorescent emission at 510 nm with the bandwidth set at 2.5 nm for both emission and excitation.

### Analysis of Intraplatelet cAMP Levels

PRP (200×10^3^/µl) was incubated both with and without forskolin (FK) for 30 s before ADP was added and the samples were incubated for 5 min at room temperature. FK stimulates AC and then increase intraplatelet cAMP levels. The reaction was stopped by the addition of 50 µl of ice-cold 30% (v/v) trichloroacetic acid. Samples were mixed and centrifuged at 6,000×g for 20 min at 4°C. Supernatants were removed and retained, and the pH was neutralized by addition of 8 mM KOH. Samples were stored at −80°C and assayed for cAMP using Amersham cAMP Biotrak EIA system (GE healthcare, UK) according to the manufacturer’s instructions.

### ESI-MS Analysis of Phospholipids

Platelets (3.6×10^7^ cells) were soaked in 200 µl of H_2_O and then sonicated for 30 s. Lipids were extracted from the lysates using the method described in Bligh and Dyer [25]. Before lipid extraction, PC with C_28∶0_ (14∶0–14∶0; m/z = 678), PE with C_28∶0_ (14∶0–14∶0; m/z = 635) and PG with C_28∶0_ (14∶0–14∶0; m/z = 666) were added to each sample as an internal standard (2 nmol per tissue) (Avanti Polar Lipids, Inc.). The analysis was performed using a 5500Q–TRAP quadrupole-linear ion trap hybrid mass spectrometer (Applied Biosystems/MDS Sciex) with an Ultimate 3000 HPLC system (Shimadzu Science, Kyoto, Japan). The extracted lipids were subjected to ESI-MS analysis by flow injection with liquid chromatography separation. The mobile phase composition was acetonitrile/methanol/water (18/11/1, v/v/v) (plus 0.1% ammonium formate (pH 6.8)) at a flow-rate of 10 µl/min. The scan range of the instrument was set at m/z 400–1000, with a scan speed of 10000 Da/s. The trap fill-time was set at 20 ms in the negative-ion mode. The ion spray voltage was set at 5500 V in the negative-ion mode. Nitrogen was used as curtain gas (setting of 20, arbitrary units) and as collision gas (set to “high”). The declustering potential was set at 60 V in the negative-ion mode. The collision energy in ESI-MS and ESI-MS/MS analyses were optimized according to the requirements of the experiment.

### Mediator Lipidomics

After 10 min of reaction, platelets were removed by centrifugation in the presence of 5 mM of ice cold EDTA and 10 µg/ml indomethacin. The platelet-free supernatant fraction was used for mediator lipidomics. Mediator lipidomics was performed as described previously [26]. Before eicosanoids extraction, 0.1 ng of prostaglandin B_2_ (GE Healthcare) was added to each sample as an internal standard. Then, 0.2% (v/v) formic acid and ethyl acetate were added to each sample before centrifugation. Supernatants were removed and used for ESI-MS/MS analysis with a 5500Q–TRAP quadrupole-linear ion trap hybrid mass spectrometer with an Ultimate 3000 HPLC system and TSKgel ODS-100V C18 column (5 µm, 4.6×150 mm; Tosoh Bioscience, Tokyo, Japan). Samples were eluted with a mobile phase composed of water/acetonitrile/formic acid (63∶37∶0.02) and acetonitrile/isoplopanol (50∶50) 73∶23, for 5 min, ramped to 30∶70 after 15 min, ramped to 20∶80 after 25 min and held for 8 min, then ramped to 0∶100 after 35 min and held for 10 min, with flow rates of 70 ml/min (0–30 min), 80 ml/min (30–33 min), and 100 ml/min (33–45 min). ESI-MS/MS analyses were conducted in negative ion mode, and eicosanoids were indicated and quantified by multiple reaction monitoring (MRM). Calibration curves (1–1000 pg) and LC retention times for each compound were established with synthetic standards.

### Bleeding Time Measurement

Bleeding time was assessed according to a previously reported method [10]. In brief, 8∼9-wks of age male mice were restrained in the upright position and their tails were transected 5 mm proximal from the tip. The remaining tail was then immersed in saline at 37°C. Bleeding time was defined as either the point at which all visible signs of bleeding from the incision stopped, or at 10 min.

### Thromboembolism Test

The tail veins of mice anesthetized with 5 mg/kg sodium pentbarbital were injected with 0.25 mg/kg collagen and 20 mg/kg of epinephrine dissolved in a buffer. Survival was evaluated 1 h after injection. Statistical analysis between WT and iPLA_2_γ-KO groups was assessed by Fisher’s exact test. The amount of collagen and epinephrine used was determined as that which induced mortality of 80%–90% in wild-type (WT) mice. For histological examination, mice were killed 2 min after injection, the heart was exposed and a 1-ml syringe with a 25-gauge needle containing EDTA powder was used to obtain about 200 µl of blood. The plasma was obtained by centrifugation of whole blood at 10,000×*g* for 15 min at 4°C, and the lungs were excised. Tissue preparations were stained with hematoxylin and eosin, and the lungs were homogenate in 1 ml of methanol. Lipids were extracted from the lysates by the method detailed in Bligh and Dyer. The thromboxane B_2_ (TXB_2_) contents of serum or lung were then used for ESI-MS/MS analysis.

### Data Analysis

Results are shown as mean±SEM from at least three individual experiments per group. Statistical analyses between WT and iPLA_2_γ-KO groups were assessed by student *t* test. *P* values less than 0.05 were considered statistically significant.

## Results

### iPLA_2_γ Expression in Murine Platelets and Morphological Features of iPLA_2_γ-Deficient Mouse Platelets

**Figure 1:**
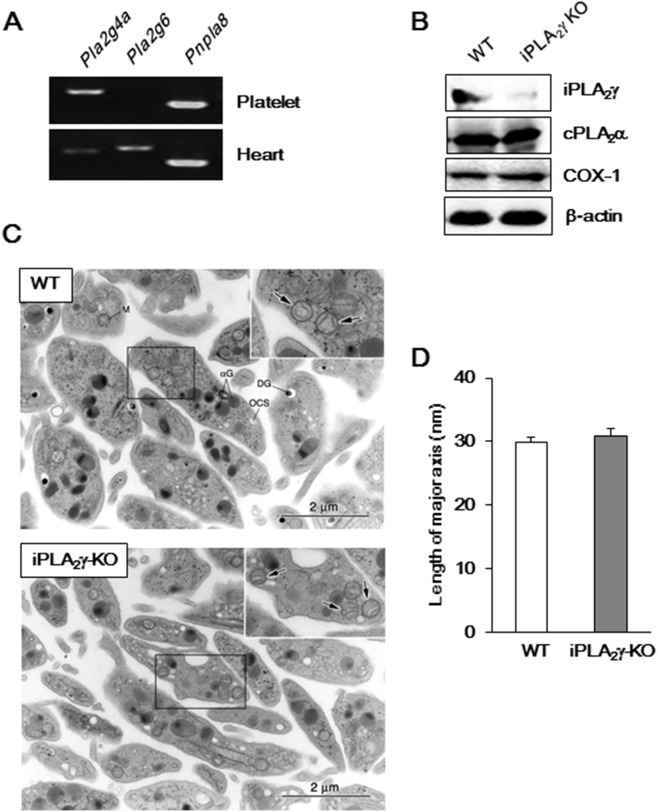
Expression of iPLA_2_γ in platelets and morphological features of iPLA_2_γ-KO mouse platelets. (A) RT-PCR analysis of cPLA_2_α (Pla2g4a), iPLA_2_β (Pla2g6) and iPLA_2_γ (Pnpla8) mRNA expressions in platelets (upper panel) and heart (lower panel). (B) Immunoblot analysis of iPLA_2_γ cPLA_2_α and COX-1 expression in WT and iPLA_2_γ-KO platelets. β-actin was used as a loading control. (C) Images of WT (upper panel) and iPLA_2_γ-KO platelet (lower panel) ultrastructure obtained by electron microscopy. M, mitochondrion; OCS, open canalicular system; αG, α-granules; DG, dense granule. Boxed areas are magnified in the upper right corners. Arrows indicate mitochondria. Representative results of at least three experiments are shown (A–C). (D) The length of the major axis of mitochondria in platelets from WT (open column) and iPLA_2_γ-KO mice (closed column). Results are the average length (nm) ±SEM (n = 3).

We first examined whether mRNAs for iPLA_2_β and iPLA_2_γ were expressed in murine platelets using the RT-PCR method (Figure 1A). Expression of mRNAs for iPLA_2_γ (*Pnpla8*) and cPLA_2_α (*Pla2g4a*) was detected, but not for iPLA_2_β (*Pla2g6*); meanwhile, the two iPLA_2_s and, to a lesser extent, cPLA_2_α were expressed in the heart used as a positive control. The absence of iPLA_2_γ protein in the iPLA_2_γ-KO mouse platelets was confirmed by western blot analysis of platelet lysates. The protein levels of COX-1 and cPLA_2_α were not significantly different between WT and iPLA_2_γ-KO mouse platelets (Figure 1B). There were no abnormalities in the platelet numbers and mean platelet volume in iPLA_2_γ-KO mice (Table 1). Furthermore, electron microscopy revealed that resting iPLA_2_γ-KO mouse platelets showed a normal discoid morphology (Figure 1C). Although previous reports showed that iPLA_2_γ-KO mice had abnormal mitochondria in skeletal muscle, myocardium and brain [23], [29], mitochondrial architecture was virtually normal in iPLA_2_γ null mouse platelets. The average length of the major axis of mitochondria in platelets was not significantly affected by iPLA_2_γ deficiency (Figure 1D).

**Table 1:** Hematological parameters of WT and iPLA_2_γ-KO mice.

	WT	iPLA_2_γ-KO
Platelets (×10^3^/µl)	598.17±101.76	530.67±114.17
White blood cells (×10^3^/µl)	10.06±0.92	8.44±0.99
Red blood cells (×10^6^/µl)	10.03±0.38	10.28±0.51
Hematocrit (%)	45.73±1.68	46.17±1.88
Hemoglobin (g/dl)	14.80±0.62	15.37±0.82
MPV (fl)	7.13±0.13	7.10±0.14

Data are mean±SEM. No abnormalities or significant differences between WT and iPLA_2_γ-KO mice were observed for hematologic parameters (n = 4–5).

MPV indicates mean platelet volume.

### Reduced ADP-dependent Aggregation of iPLA_2_γ-deficient Mouse Platelets

**Figure 2:**
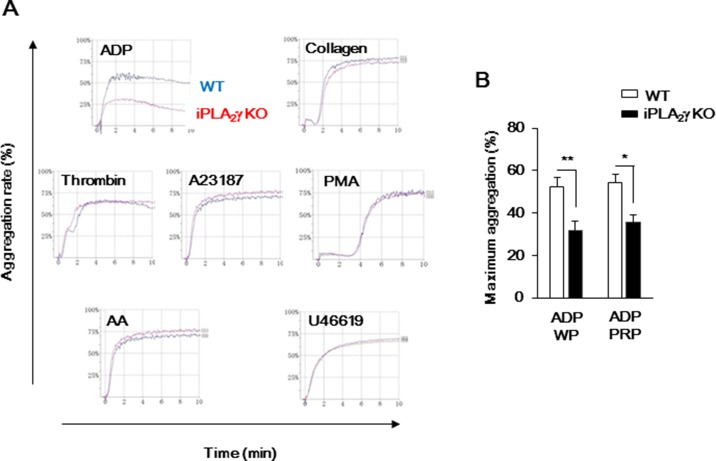
iPLA_2_γ deficiency inhibits platelet aggregation in response to ADP stimulation. (A) Representative results from aggregometry testing. Washed platelets from WT (blue) or iPLA_2_γ-KO (red) mice were stimulated with the indicated agonists (ADP (10 µM), collagen (1 µg/ml), thrombin (0.1 U/ml), A23187 (5 µM), PMA (10 nM), AA (100 µM) or U46619 (5 µM)), and then light transmission was recorded on an aggregometer. (B) Bar graphs indicate results obtained by aggregometry tests. Washed platelets (WP) or platelets in PRP (200×10^3^/µl) from WT or iPLA_2_γ-KO mice were stimulated with ADP (10 µM), and then light transmission was recorded on an aggregometer. Results are given as the mean percentage of maximum aggregation±SEM (n = 6–10). *P<0.05 and **P<0.01 between iPLA_2_γ-KO and WT.

**Figure 3:**
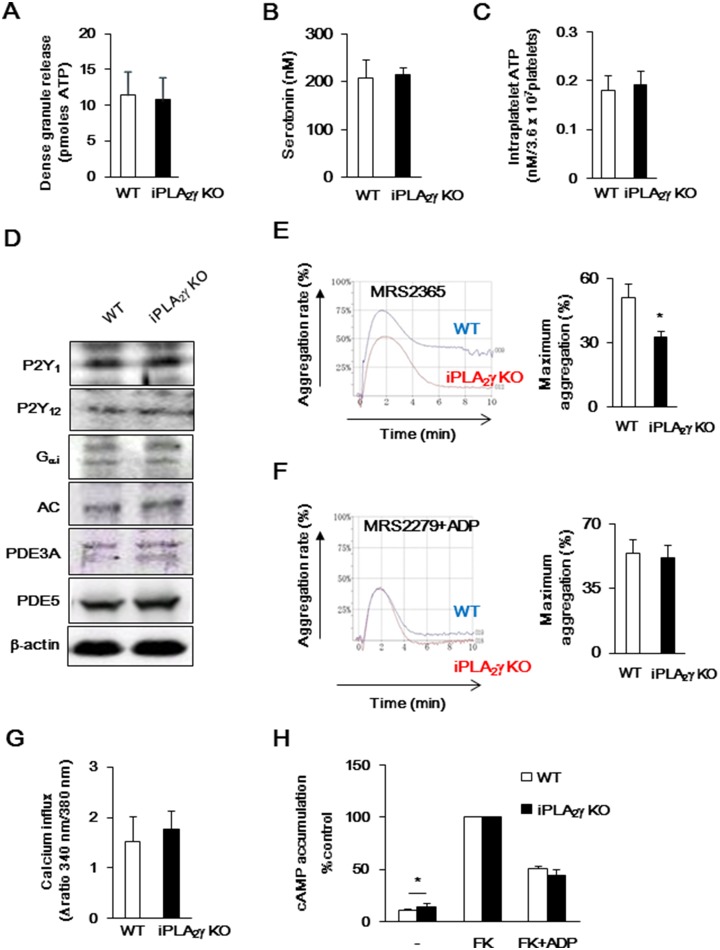
P2Y receptors-mediated signaling of iPLA_2_γ deficient platelets. (A and B) Dense granule secretion was evaluated by measuring ATP (A) and serotonin (B) release after the ADP aggregation of WT (open columns) or iPLA_2_γ-KO (closed columns) platelets. Results are expressed as the amounts of ATP (n = 10–13) and serotonin (n = 3–4) released by 3.6×10^7^ from platelets. (C) Measuring intraplatelet ATP levels of resting state of WT (open columns) or iPLA_2_γ-KO (closed columns) platelets (n = 5). (D) Protein expression of P2Y_1_, P2Y_12_, G_αi_, AC and PDEs of platelets from WT and iPLA_2_γ-KO mice. β-actin was used as a loading control. Representative results of at least three experiments are shown. (E and F) PRP from WT (blue) or iPLA_2_γ-KO (red) mice were stimulated with P2Y_1_ agonist (MRS2365) (10 µM) (E) or P2Y_1_ antagonist (MRS2279) (10 µM) plus ADP (10 µM) (F), and then light transmission was recorded on an aggregometer. Left panel indicates representative results from aggregometry testing. Right graphs indicate results obtained by aggregometry tests. Results are given as the mean percentage of maximum aggregation±SEM (n = 6–10). *P<0.05 between iPLA_2_γ-KO and WT. (G) Ca^2+^ influx of WT (open columns) or iPLA_2_γ-KO (closed columns) platelets in response to ADP (10 µM). Results are given as mean±SEM (n = 8). (H) PRP (200×10^3^/µl) from WT (open columns) or iPLA_2_γ-KO (closed columns) was incubated both with and without forskolin (FK; 5 µM) for 30 s before ADP (10 µM) was added and the samples were incubated for 5 min at room temperature. Results are given as the mean±SEM (n = 5). *P<0.05 between iPLA_2_γ-KO and WT.

As shown in Figure 2, functional studies of platelets from iPLA_2_γ-KO mice, compared to WT mice, revealed that ADP-induced aggregation was reduced, whereas aggregation in response to other platelet activators, including collagen, thrombin, Ca^2+^-ionophore (A23187), PMA, AA and TXA_2_ receptor (TP) agonist (U46619) were similar between iPLA_2_γ-KO and WT platelets. Even when PRP was stimulated with ADP, platelet aggregation was also reduced by iPLA_2_γ deficiency. The release of the contents in platelet-dense granules has been thought to play an important role in perpetuating the aggregation response [1], [3]. We therefore investigated the effect of iPLA_2_γ deletion on ADP-induced dense granule release by quantifying liberated ATP and serotonin. In response to ADP, platelets from iPLA_2_γ-KO mouse secreted ATP and serotonin to levels comparable to those from WT platelets (Figure 3A and B). Moreover, intracellular ATP levels were not significantly different between WT and iPLA_2_γ-deficient mouse platelets (Figure 3C). These results indicated that iPLA_2_γ deletion affected neither ADP-induced dense granule release nor cellular ATP synthesis, which is consistent with normal mitochondrial morphology in the iPLA_2_γ-KO mice (Figure 1C).

ADP induces platelet aggregation through two G-protein coupled receptors --- G_q_-coupled P2Y_1_, and G_i_-coupled P2Y_12_ [28], [29]. The protein levels of P2Y_1_ and P2Y_12_ in iPLA_2_γ-deficient mouse platelets were similar to those in WT platelets (Figure 3D). Like ADP-induced platelet aggregation, P2Y_1_ agonist MRS2365-induced aggregation was also reduced by iPLA_2_γ deficiency (Figure 3E). On the other hand, when platelets were pretreated with P2Y_1_ antagonist MRS2279 and then stimulated with ADP, platelet aggregation from iPLA_2_γ-KO mouse was similar to that of WT platelets (Figure 3F). These results indicated that iPLA_2_γ is involved in P2Y_1_-mediated platelet aggregation by ADP stimulation, not P2Y_12_-. P2Y_1_ receptor stimulation increases intracellular Ca^2+^ levels by phospholipase Cβ activation, and P2Y_12_ receptor stimulation results in G_αi_-mediated inhibition of AC [28], [29]. We further examined the effects of iPLA_2_γ deficiency on ADP-induced second messenger signaling, but our analysis revealed that there was little difference in second messenger signaling between WT and iPLA_2_γ-deficient mouse platelets. ADP-induced increment in intracellular Ca^2+^ levels was not significantly affected by iPLA_2_γ deficiency (Figure 3G). Notably, intracellular cAMP levels in non-treated iPLA_2_γ-deficient mouse platelet were significantly higher than in WT platelets (Figure 3H), although the protein levels of AC, PDE3A, PDE5 and G_αi_ subunit in iPLA_2_γ-deficient mouse platelets were similar to those in WT platelets (Figure 3D). However, the increased cAMP level in FK-treated iPLA_2_γ-deficient mouse platelets was decreased by ADP stimulation to a level similar to that in WT platelets (Figure 3H).

### Lipidomics of Platelets

**Figure 4:**
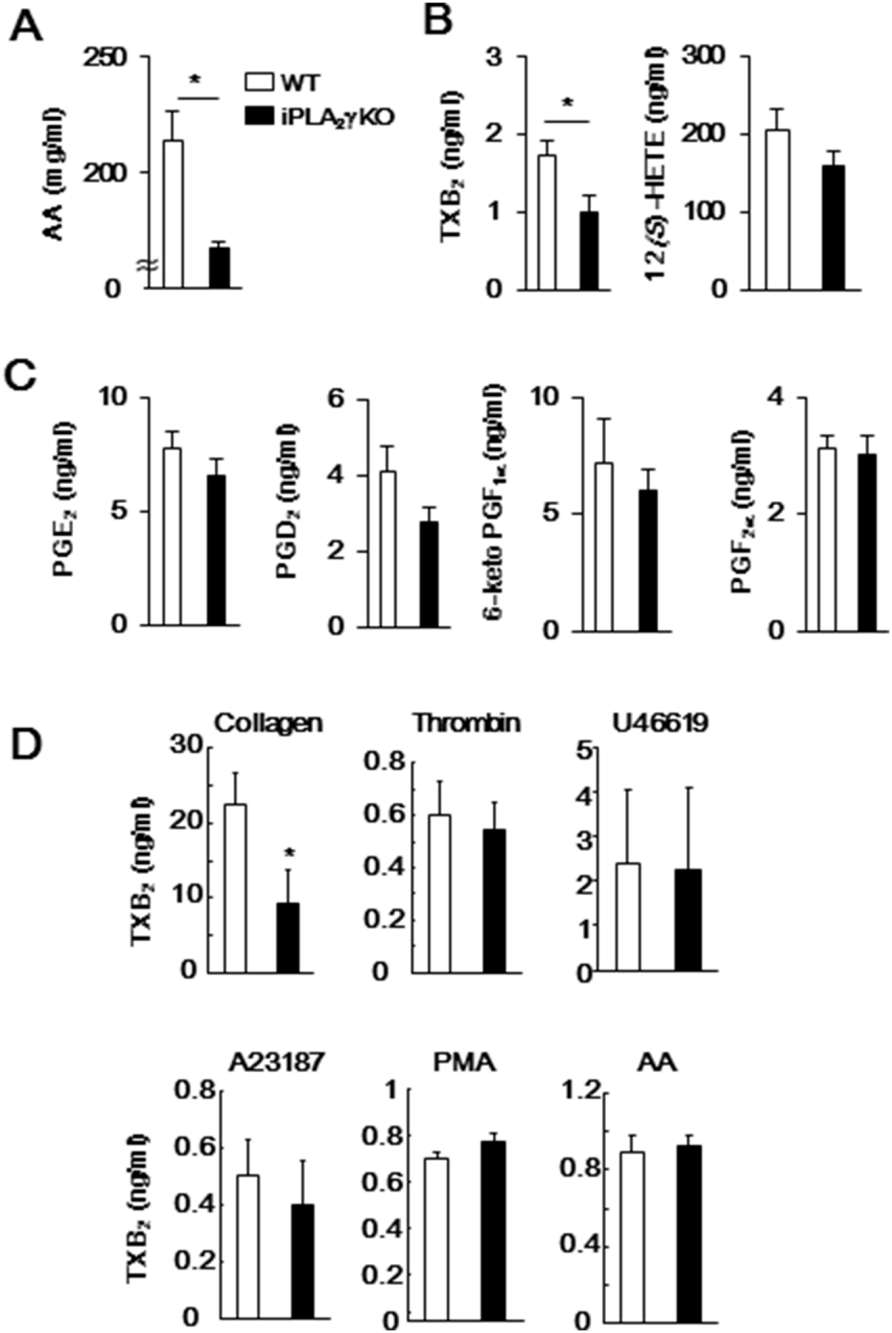
Amounts of lipid mediators produced from WT and iPLA_2_γ-KO mouse platelets after ADP stimulation. Levels of (A) AA, (B) TXB_2_ (a TXA_2_ metabolite) and 12*(S)*-HETE, and (C) PGE_2_, PGD_2_, 6-keto prostaglandin F_1α_ (6-ketoPGF_1α_) (a prostacyclin metabolite) and prostaglandine F_2α_ (PGF_2α_) in supernatants from WT (open columns) or iPLA_2_γ-KO (closed columns) platelets stimulated with ADP (10 µM). (D) Levels of TXB_2_ in supernatants from WT (open columns) or iPLA_2_γ-KO (closed columns) mouse platelets stimulated with collagen (1 µg/ml), thrombin (0.1 U/ml), A23187 (5 µM), PMA (10 nM), AA (100 µM) or U46619 (5 µM). Results are given as mean±SEM (n = 3–9). *P<0.05 between iPLA_2_γ-KO and WT.

We further examined the effects of iPLA_2_γ deletion on AA release and production of AA metabolites by ADP-activated platelets using ESI-MS/MS analysis. Release of AA and TXA_2_ (measured as its stable analog, TXB_2_) from iPLA_2_γ-deficient mouse platelets was significantly reduced compared to that of WT platelets (Figure 4A and B). Although there were no significant differences between genotypes, the levels of other AA metabolites, such as 12*(S)*-HETE, PGE_2_ and PGD_2_ also tended to be lower in iPLA_2_γ-KO mouse platelets than in WT mouse platelets (Figure 4B and C). In addition, collagen-induced TXA_2_ generation was decreased in iPLA_2_γ-deficient mouse platelets, although TXA_2_ generation in response to thrombin, A23187, PMA, AA and U46619 were not significantly different (Figure 4D).

**Figure 5:**
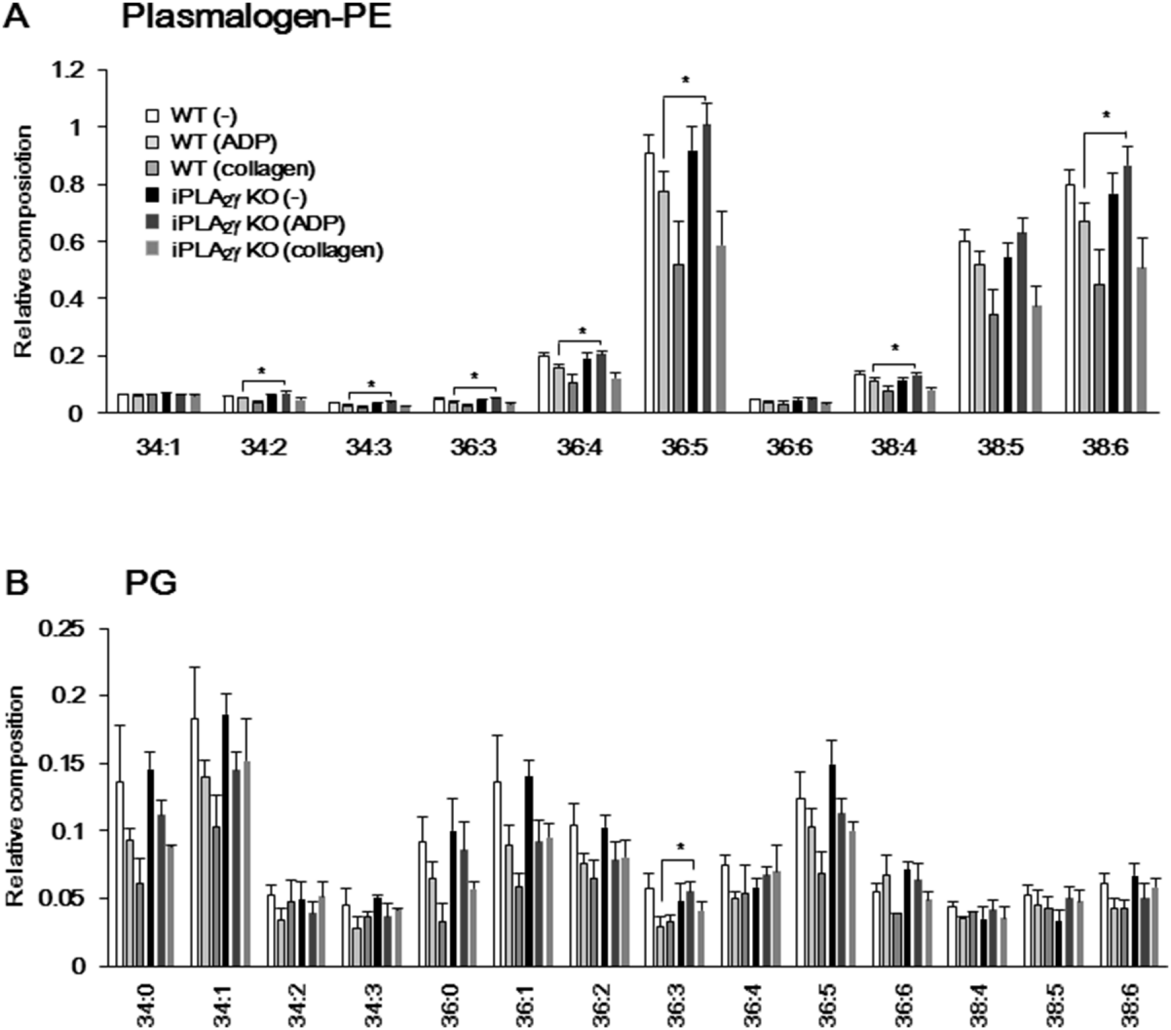
ESI/MS analysis of plasmalogen-PE and PG species in WT and iPLA_2_γ-KO mouse platelets. Total lipids were extracted from resting or ADP (10 µM)-stimulated platelet lysates and then subjected to ESI/MS analysis of PG and PE. Levels of (A) plasmalogen-PE and (B) PG in resting state of WT (–) or iPLA_2_γ-KO platelets (–), or ADP-stimulated WT (ADP) or iPLA_2_γ-KO platelets (ADP), and collagen (1 µg/ml)-stimulated WT (collagen) or iPLA_2_γ-KO platelets (collagen) (n = 7). Results are given as mean±SEM. *P<0.05 between iPLA_2_γ-KO and WT.

To assess the contribution of iPLA_2_γ to stimulant-dependent phospholipid hydrolysis in platelets, we performed ESI/MS lipidomics analysis. Interestingly, some specific species of phospholipids, namely alkenyl forms of PE (plasmalogen PE) and PG bearing C_18∶2_ (linoleic acid) or C_20∶4_ (AA) at the *sn-2* position, were selectively decreased by ADP stimulation in WT platelets, whereas the decrease of these species was not observed in platelets from the iPLA_2_γ-KO mouse (Figure 5). As shown in Figure 5A, many, if not all, of the subclasses of plasmalogen PE, such as those with C_34∶2_ (C_16∶0_ and C_18∶2_), C_34∶3_ (C_16∶1_ and C_18∶2_), C_36∶3_ (C_18∶1_ and C_18∶2_), C_36∶4_ (C_16∶0_ and C_20∶4_), C_36∶5_ (C_16∶1_ and C_20∶4_), C_38∶4_ (C_18∶0_ and C_20∶4_), and C_38∶6_ (C_18∶2_ and C_20∶4_), were decreased by ADP stimulation in WT platelets, whereas the decrease of these species was not observed in iPLA_2_γ-deficient mouse platelets. The plasmalogen PE subclasses with C_36∶4_, C_36∶5_, C_38∶4_, and C_38∶6_ were confirmed to contain AA, because a molecular ion peak of *m/z* 303 ( = C_20∶4_), in addition to that of the corresponding parent ion, was mainly detected on the MS/MS.

**Figure 6:**
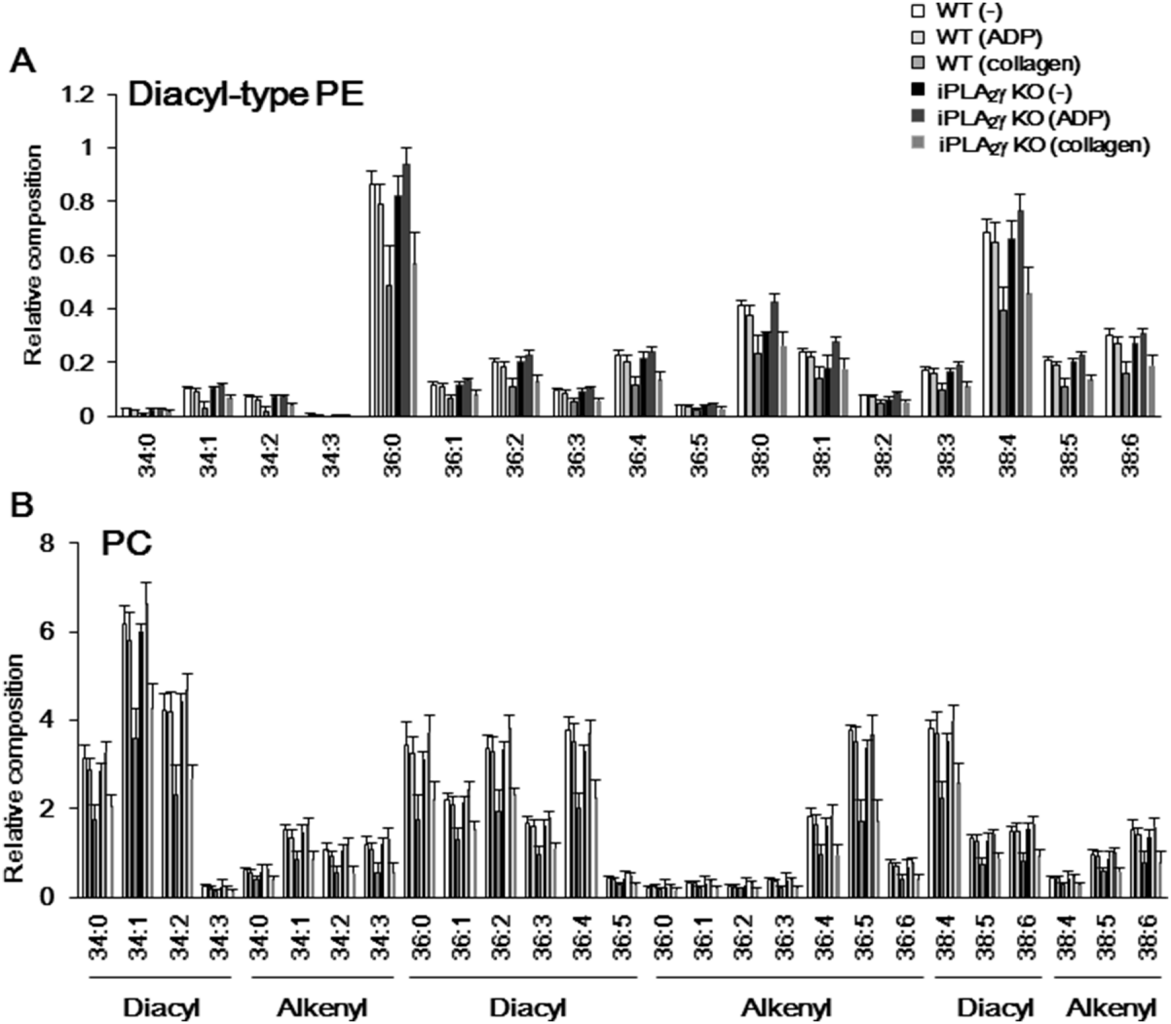
ESI/MS analysis of diacyl-type PE and PC species in WT and iPLA_2_γ-KO mouse platelets. Total lipids were extracted from resting or ADP (10 µM)-stimulated platelet lysates and then subjected to ESI/MS analysis of diacyl-type PE and PC. Levels of (A) diacyl-type PE and (B) PC in resting state of WT (–) or iPLA_2_γ-KO platelets (–), or ADP-stimulated WT (ADP) or iPLA_2_γ-KO platelets (ADP), and collagen (1 µg/ml)-stimulated WT (collagen) or iPLA_2_γ-KO platelets (collagen) (n = 7). Results are given as mean±SEM.

On the other hand, all most all of phospholipid subclasses were decreased by collagen stimulation and there was little difference in collagen-induced phospholipid hydrolysis between WT and iPLA_2_γ-KO platelets. As shown in Figure 5B, iPLA_2_γ-deficient mouse platelets in a resting state showed a tendency to contain lower PG subclasses with C_36∶3_, C_36∶4_, C_38∶4_,and C_38∶5_ than did WT mouse platelets. These PG species were decreased in WT mouse platelets, but not in iPLA_2_γ-deficient mouse platelets after ADP or collagen stimulation. By contrast, there was no significant difference in composition of the diacyl forms of PE, or in essentially all of the molecular species of PC between WT and iPLA_2_γ-KO (Figure 6). These data suggest that iPLA_2_γ mainly catalyzes the hydrolysis of plasmalogen PE and PG-bearing C_18∶2_ and C_20∶4_ in a resting state, or ADP-activated platelets, and that these released AA are metabolized to eicosanoids, including TXA_2_.

### Impaired Hemostasis and Decreased Susceptibility to Thromboembolism in iPLA_2_γ-deficient Mice

**Figure 7:**
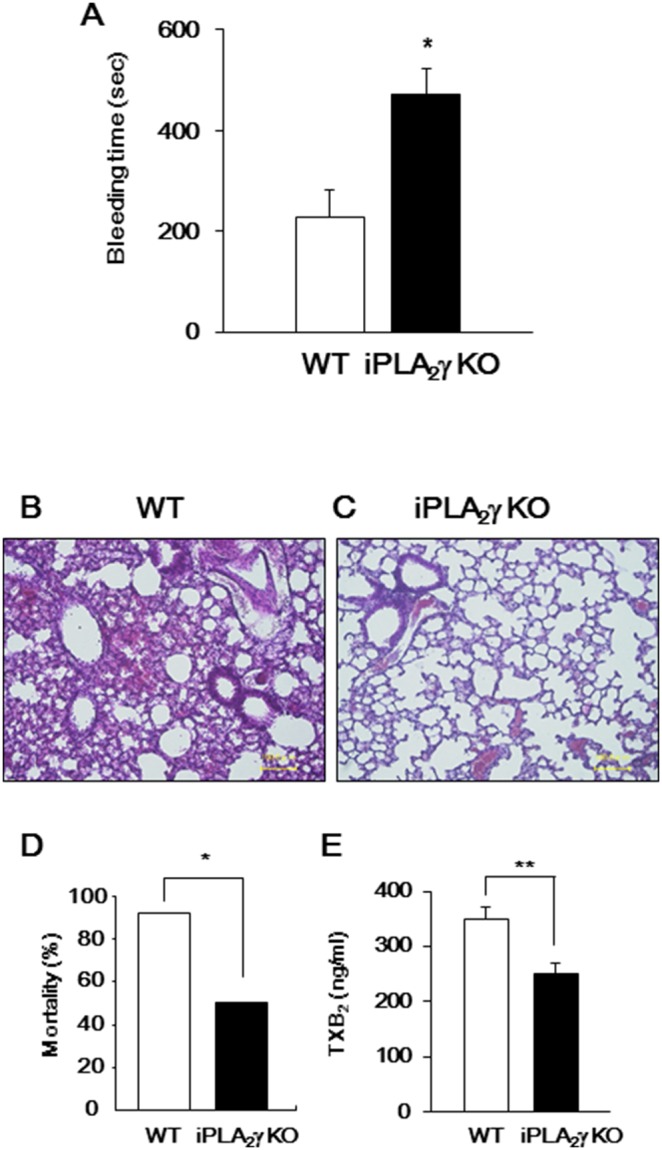
Impaired hemostasis and thrombus formation in iPLA_2_γ-KO mice. (A) Bleeding times for WT (open column; n = 9) and iPLA_2_γ-KO (closed column; n = 11) mice. Data are mean±SEM. **P<0.01 between iPLA_2_γ-KO and WT. (B–D) Thrombotic challenge in WT (n = 13) and iPLA_2_γ-KO mice (n = 12). (B and C) Histological examination of lungs from (B) WT and from (C) iPLA_2_γ-KO mice killed 2 min after injection of 0.25 mg/kg collagen and 20 mg/kg epinephrine mixure. Representative results of at least three experiments are shown. (D) Data represent percentage of deaths within 1 hr after injection of collagen and epinephrine mixure. *P* values were determined by Fisher’s exact test: *P<0.05 between iPLA_2_γ-KO (n = 12) and WT (n = 13). (E) Serum TXB_2_ content after injection of collagen and epinephrine mixture. Data are mean±SEM. *P<0.05 between iPLA_2_γ-KO (n = 3) and WT (n = 6).

To delineate the role of iPLA_2_γ in platelet hemostasis and thrombus formation *in vivo*, a tail-bleeding time assay and thromboembolism test were performed. We first found that bleeding times were significantly longer in iPLA_2_γ-KO mice than in gender-matched WT mice (Figure 7A).

Next, WT and iPLA_2_γ-KO mice were intravenously injected with a mixture of collagen and epinephrine, which causes lethal pulmonary thromboembolism. This mouse model is often used to assess ADP-induced platelet activation *in vivo*. In fact, this model had revealed that both P2Y_1_ genetic deletion and antagonists increased resistance to thrombosis *in vivo* [30], [31]. As shown in Figure 7B, histological examination showed marked thrombus formation in the arterioles of the lung in WT mice. Alveolar hemorrhage was also observed in broad areas, frequently accompanying massive pulmonary thrombosis. In contrast, scarce evidence of such thrombus formation or alveolar hemorrhage was found in the lung from iPLA_2_γ-KO mice (Figure 7C). Only 10% of WT mice survived, whereas 50% of iPLA_2_γ-KO mice were still alive 60 min after the challenge (Figure 7D). TXB_2_ levels in the serum from iPLA_2_γ-KO mice after injection were significantly lower than those from WT mice (Figure 7E). These results suggest that iPLA_2_γ plays an important role in *in vivo* TXA_2_ production accompanied by thrombus formation.

## Discussion

PLA_2_ plays a central role in platelet activation by hydrolysis of membrane phospholipids in response to a variety of stimuli. Previous studies have shown that, among several different PLA_2_ enzymes, cPLA_2_α is critical for platelet activation, even though other PLA_2_(s) may also be involved [10]. The present study has revealed that iPLA_2_γ, one of the Ca^2+^-independent intracellular PLA_2_ enzymes, represents the missing link; it is also responsible for stimulus-dependent AA release and functions as a key enzyme in platelet aggregation *in vitro* and thrombus formation *in vivo*. Only the metabolic roles of iPLA_2_γ have thus far been highlighted *in vivo* [23], [27], [32]. This is the first demonstration that iPLA_2_γ has a previously unrecognized homeostatic role in a particular lineage of hematopoietic cells, namely platelets.

When WT platelets were stimulated with ADP, breakdown of PE (plasmalogen-type) and PG-bearing AA at the *sn*-2 position was obvious (Figures 5). In sharp contrast, the amounts of these PE subclasses were unaffected by ADP stimulation in iPLA_2_γ-deficient platelets. In addition to the release of AA, the production of TXA_2_ was also reduced by iPLA_2_γ deficiency (Figure 4). These results suggest that in mouse platelets, iPLA_2_γ is activated in ADP-stimulated platelets and selectively hydrolyzes AA-containing plasmalogen-PE to release AA, leading to the production of TXA_2_. The production of other AA metabolites, such as 12*(S)*-HETE, PGE_2_ and PGD_2_ also tended to be reduced in iPLA_2_γ-KO mouse platelets. iPLA_2_γ might be preferentially coupled with COX-1-TXA_2_ synthase pathway but a portion of iPLA_2_γ-liberated AA might be utilized by the other metabolic pathway. By comparison, in cPLA_2_α-deficient platelets, ADP-induced TXA_2_ generation remained entirely intact and collagen-induced TXA_2_ generation was reduced by only half [10]. This implies that, at least under these particular stimuli, iPLA_2_γ could account largely, if still not solely, for the TXA_2_ pool produced independently of cPLA_2_α. It should be noted that the levels of several PG subclasses tended to be lower in iPLA_2_γ-deficient platelets than in WT platelets, even in the absence of stimuli (Figure 5A). The lower levels of these PG subclasses might lead to reduction in hydrolysis of PG in response to collagen, as well as to ADP. Cardiolipin and its precursor PG are mostly confined to mitochondrial membranes. Levels of both cardiolipin and PG were reduced in the heart, brain and skeletal muscle of the iPLA_2_γ-deficient mice [23], [29], [32]. iPLA_2_γ has been reported to be localized in the mitochondria, peroxisomes and ER of several cells [20], [27]. In mouse platelets, iPLA_2_γ may be involved in the maintenance of mitochondrial membranes through membrane remodeling, as well as stimulus-dependent hydrolysis of phospholipids. However, mitochondrial morphology and activity (as monitored by ATP synthesis) appeared to be intact in iPLA_2_γ-deficient platelets, underscoring a difference from the profound effects of iPLA_2_γ deficiency on mitochondrial homeostasis in brain, heart and skeletal muscle [22], [29], [32].

Selective hydrolysis of plasmalogen-type phospholipids in response to thrombin, collagen or U46619 has also been reported in human platelets [12]. Human platelets express both iPLA_2_β and iPLA_2_γ; in that they differ from mouse platelets, in which iPLA_2_γ is dominant. Amounts of arachidonylated plasmenylcholine and plasmalogen species were significantly decreased by thrombin stimulation in human platelets. It was also shown that pretreatment with the iPLA_2_γ-specific inhibitor *R*-BEL inhibited these thrombin-stimulated phospholipid hydrolyses more effectively than iPLA_2_β inhibitor *S*-BEL [12]. Our previous experiments found that overexpression of iPLA_2_γ caused reduction in AA-containing plasmalogen subclasses in HEK293 cells [20]. Thus, activated iPLA_2_γ appears to selectively hydrolyze AA-containing plasmalogen-type phospholipids not only in mouse platelets but also in several tissues and cells including human platelets. In mouse platelets, ADP- or collagen-induced TXA_2_ generation was reduced by iPLA_2_γ deficiency, although its generation in response to other stimuli such as thrombin was unaffected (Figure 4). There was no difference in hydrolysis of plasmalogen PE in response to collagen between WT and iPLA_2_γ-deficient platelets (Figure 5B). Agonist-dependent activation mechanism of iPLA_2_γ might be different between human and mouse platelets.

Furthermore, in this study we found that among the agonists tested, only ADP-induced platelet aggregation was reduced by iPLA_2_γ deficiency (Figure 2). Although TXA_2_ generation induced by ADP or collagen was reduced (Figure 4), aggregation in response to stimuli other than ADP, including collagen, was not affected. It has been reported that there are TXA_2_-dependent and -independent pathways in platelet aggregation [33], [34]. As iPLA_2_γ-deficient platelets could fully aggregate in response to AA and U46619 (Figure 2), the TXA_2_-dependent aggregation pathway was not affected by iPLA_2_γ deficiency. Our experiments using the P2Y_1_ agonist and antagonist showed that iPLA_2_γ is involved in P2Y_1_-mediated platelet aggregation (Figure 3D and E). Although P2Y_1_ is coupled with G_q_ and its activation leads to Ca^2+^ mobilization [29], iPLA_2_γ deficiency did not affect ADP-induced increment in intracellular Ca^2+^ levels through G_q_-coupled P2Y_1_ receptor (Figure 3G) Intraplatelet crosstalk between iPLA_2_γ activation and Ca^2+^ mobilization may regulate ADP-induced aggregation.

It is noteworthy that mice lacking iPLA_2_γ have prolonged bleeding times and are resistant to thromboembolism induced by injection of epinephrine and collagen, as is the case with cPLA_2_α-deficient mice [10]. These results indicate that iPLA_2_γ plays a critical role in platelet hemostasis and thrombus formation *in vivo*, although iPLA_2_γ deletion did not affect *in vitro* platelet aggregation in response to platelet activators other than ADP. As inappropriate thrombus formation could lead to acute coronary syndromes and progression of atherosclerotic disease, antithrombotic drugs are used for prevention and therapy for these diseases. Three classes of inhibitors of platelet aggregation have demonstrated substantial clinical benefits. Aspirin acts by irreversibly inhibiting COX-1, and therefore blocking the synthesis of TXA_2_. The indirect-acting (ticlopidine, clopidogrel, prasugrel) and direct-acting (ticagrelor) antagonists of P2Y_12_ block the thrombus-stabilizing activity of ADP. Parenteral GPIIb/IIIa inhibitors directly block platelet-platelet interactions. Despite well-established benefits, all of these antiplatelet agents have important limitations. iPLA_2_γ has proven to be a potential target for antithrombotic drug development.
